# Recovery of insulin/IGF‐1/GLP‐1 signaling protects the brain against cardiometabolic and neurodegenerative diseases

**DOI:** 10.1002/alz.89823

**Published:** 2025-01-09

**Authors:** Ana Isabel Duarte

**Affiliations:** ^1^ iCBR ‐ Coimbra Institute for Clinical and Biomedical Research, Faculty of Medicine, University of Coimbra, Coimbra, Coimbra, Portugal; Institute of Pharmacology and Experimental Therapeutics, Faculty of Medicine, University of Coimbra, Coimbra, Coimbra, Portugal; CIBB ‐ Center for Innovative Biomedicine and Biotechnology, University of Coimbra, Coimbra, Coimbra, Portugal; Institute of Interdisciplinary Research (IIIUC), University of Coimbra, Coimbra, Coimbra, Portugal; CNC‐UC ‐ Center for Neuroscience and Cell Biology, University of Coimbra, Coimbra, Coimbra Portugal

## Abstract

**Background:**

Cardiometabolic diseases, such as type 2 diabetes, hypertension, dyslipidemia or obesity, constitute major causes of mortality and morbidity worldwide, especially among middle‐aged individuals. The increasing incidence and association with aging and lifestyle, render the cardiometabolic diseases a societal concern. This is further reinforced by their association with an increased risk of cognitive impairment and neurodegenerative diseases (namely dementia and Alzheimer’s disease (AD)).

Given the poor knowledge on the precise crosslinking mechanisms between cardiometabolic and neurodegenerative diseases, we hypothesize that recovering insulin/IGF‐1/GLP‐1 signaling protects against brain and cognitive injury upon cardiometabolic diseases, ultimately preventing neurodegenerative diseases. We aimed to study the impact of recovering brain insulin/IGF‐1/GLP‐1 signaling on brain and cognitive function in animal models of cardiometabolic and neurodegenerative diseases.

**Methods:**

We used rodent models of type 2 diabetes, high cardiovascular risk, AD and Huntington’s disease (HD), peripherally administered with commercially available GLP‐1 mimetics or anti‐dyslipidemia drugs, to analyze the features of cardiometabolic and neurodegenerative diseases, markers of oxidative stress, neuroinflammation, (mitochondrial) metabolism and autophagy/mitophagy, together with the motor and cognitive performance.

**Results:**

Our studies suggest that recovering cerebral insulin/IGF‐1/GLP‐1, either by peripherally administered incretins or novel anti‐dyslipidemia drugs, may protect against oxidative stress, inflammation, dysregulated metabolic and autophagic mechanisms, ultimately counteracting motor and cognitive impairment and neurodegeneration/death in rodent models of cardiometabolic and neurodegenerative diseases (namely type 2 diabetes, AD, HD, high cardiovascular risk).

**Conclusions:**

Administration of anti‐type 2 diabetes or anti‐dyslipidemia drugs that recover cerebral insulin/IGF‐1/GLP‐1 signaling may constitute a promising preventive/therapeutic strategy against persistent brain injury associated with cardiometabolic and neurodegenerative diseases. However, this issue deserves to be further clarified.

Funded by: European Regional Development Fund, through the *Centro2020 Operational Programme* (Projects *CENTRO‐01‐0145‐FEDER‐000012 (HealthyAging2020)*, *POCI‐010145‐FEDER‐016577, POCI‐01‐0145‐FEDER‐029391* (*Mito4ALS*)); by *COMPETE 2020* ‐ Operational Programme for Competitiveness and Internationalization; by Portuguese funds via FCT – Fundação para a Ciência e a Tecnologia, through the projects *PTDC/SAU‐TOX/117481/2010, POCI‐01‐0145‐FEDER‐029391, PTDC/MED‐FAR/29391/2017, UIDB/04539/2020, UIDP/04539/2020, LA/P/0058/2020*; by Santander‐Totta & Faculty of Medicine, University of Coimbra (*PEPITA 2018*); by the European Social Fund, though the contracts *DOI: 10.54499/DL57/2016/CP1448/CT0006* and *EU HORIZON‐WIDERA‐2022‐ACCESS‐04‐01 Excellence Hubs/CHAngeing/101087071/II0347.01* of AID.

